# CEP290 is required for photoreceptor ciliogenesis and other cilia related functions

**DOI:** 10.1186/2046-2530-1-S1-P98

**Published:** 2012-11-16

**Authors:** RA Rachel, EA Yamamoto, M Dewanjee, J Munasinghe, HL May-Simera, L Dong, A Swaroop

**Affiliations:** 1 NIH/National Eye Institute, USA; 2 NIH/Neurological Diseases & Stroke, USA

## 

Mutations in *CEP290*, which encodes a centrosomal/cilia protein, result in a broad range of human ciliopathies from isolated Leber congenital amaurosis to lethal Meckel-Gruber syndrome. By light microscopy, CEP290 localizes to the ependymal cells lining the lateral ventricles, to respiratory airways, and to ciliated cells of the kidney. By immunoEM in photoreceptors, CEP290 localizes to ciliary microtubules of the connecting cilium. We generated a null allele of *Cep290* in mice by replacing exons 1-4 of the *Cep290* gene with a lacZ-neo cassette and analyzed *Cep290*-knockout mouse phenotypes. *Cep290ko/ko* mice develop hydrocephalus and most (80-100%, strain-dependent) die by one month. Both C57BL/6 and 129SvJ backgrounds reduce viability of *Cep290ko/ko* mice. High-resolution MRI of the brain demonstrates enlarged ventricles and other morphological abnormalities. By SEM ependymal cells in the *Cep290ko/ko* mice have reduced numbers of unorganized cilia, in contrast to the tufts of cilia lining the WT ventricular epithelium. Knockout mice having subclinical hydrocephalus survive and reproduce normally. Photoreceptors fail to generate connecting cilia or outer segments in *Cep290ko/ko* mice, although basal bodies and associated microtubule assemblies are found. The presence of basal bodies and microtubule rings without connecting cilia or outer segments indicates that CEP290 is essential for ciliogenesis in photoreceptors. Most photoreceptors die between P14 and P28. *Cep290ko/*+ mice do not have retinal degeneration or hydrocephalus, demonstrating haplosufficiency. Our results indicate that CEP290 plays a critical role in ciliogenesis and cilia function in subsets of neurons and have begun to shed light on underlying mechanisms in ciliopathies.

